# Pantothenate kinase-associated neurodegeneration (PKAN) in a child with Down syndrome. A case report and follow-up with MRI

**DOI:** 10.1259/bjrcr.20150011

**Published:** 2015-07-07

**Authors:** A Hakim, C Rozeik, M Fedorcak

**Affiliations:** ^1^ Radiology Department, Lörrach District Hospital, Lörrach Germany; ^2^ Centre of Paediatrics and Youth Medicine, St. Elizabeth Hospital, Lörrach Germany

## Abstract

Neurodegeneration with brain iron accumulation (NBIA) is a subtype of inherited metabolic disorders. It includes pantothenate kinase-associated neurodegeneration (PKAN), which is a rare autosomal recessive disorder caused by the mutation of pantothenate kinase 2-gene (PANK2). It affects the deep grey matter nuclei causing progressive extrapyramidal motor impairment.1 We present a case of genetically proven PKAN in a child with mosaic trisomy 21 in which T2- hyperintensity of the basal ganglia was seen followed by the classic eye-of-the-tiger sign in a follow-up MRI.

## Case report

**Figure 1.**
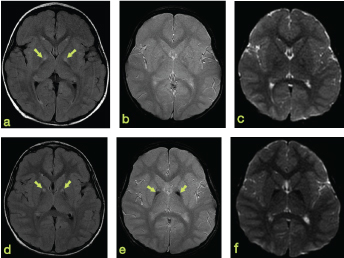
MRI of the brain at the age of 27 months (a–c) and at the age of 5 years (d–f). (a,d) Axial fluid-attenuated inversion recovery, (b,e) axial *T*_2_* GRE, (c,f) axial b0 of diffusion weighted imaging. The upper row at the age of 27 months showing bilateral *T*_2_ hyperintensity in the medial globus pallidus (arrows in a) and no remarkable signal changes in the *T*_2_* images at this age. In the follow-up MRI at the age of 5 years (lower row), there is obvious new hypointense signal in the *T*_2_* GRE in the lateral global pallidus bilaterally (arrows in e) with hyperintensity medially (arrows in d). GP, GRE, gradient recalled echo.

A daughter of unrelated healthy parents was referred to our radiology department at the age of 27 months owing to afebrile seizures. MRI showed a symmetrically increased *T*_2_ signal intensity in the basal ganglia of undetermined aetiology (Figure 1), for which a long-term follow-up was advised. Shortly after the MRI, a karyotyping was performed and showed 47, XX, +21 in 8 of 20 cells (mosaic trisomy 21).

Furthermore, the parents had noticed a slight psychomotor developmental delay in their daughter since her first year of life. She did not start walking until the age of 2 years and speech was also delayed; furthermore, there was regression of her previously learnt vocabulary. By her fourth year, dystonia was first noticed in the form of contractions of both hands. Difficulty in swallowing was also noticed early on.

**Figure 2.**
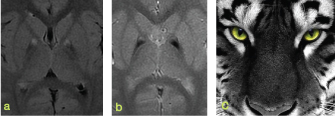
Magnified images of the basal ganglia of the MRI performed at the age of 5 years, showing a hyperintense signal at the medial globus pallidus in axial fluid-attenuated inversion recovery (a) and a hypointense signal at the lateral globus pallidus , especially at the *T*_2_* sequence (b). The combination of these signals is known as the eye-of-the-tiger sign (c). Source of image (c): http://heroschool.us/youve-got-the-eye-of-the-tiger/

A follow-up MRI was performed at the age of 5 years and showed a persistent bilateral hyperintense signal in the medial globus pallidus (GP) (Figure 1). A surrounding hypointense signal, especially in *T*_2_* gradient recalled echo (GRE), not seen on the previous MRI, was newly detected (Figure 1) as well as the combined signals in the basal ganglia resembling the eye-of-the-tiger (Figure 2). Neither diffusion restriction nor enhancement was noticed.

Owing to this characteristic appearance of the eye-of-the-tiger pattern, the radiological diagnosis strongly suggested PKAN. A molecular genetic test was performed and confirmed the diagnosis by the presence of missense mutation in the *PANK2* gene [c.683T>C (p. F228S) in exon 4 and c.1561G>A (p. G521R) in exon 8]. Genetic testing of the mother was then performed and showed her to be an asymptomatic carrier (heterozygote) by presence of the same mutation, c.683T>C (p. F228S).

At present, the child is being managed with anti-epilepsy medication. Iron chelation therapy with deferiprone is scheduled.

## Discussion

In 1922, a disease with extrapyramidal symptoms, in which the basal ganglia were involved, was described. Currently, this disease is usually classified as a neurodegenerative disorder with brain iron accumulation (NBIA) and includes 10 subtypes that represent different mutations. The *PANK2* gene, which is found in chromosome 20p12.3–13, and its mutation is responsible for PKAN being the most frequent form of NBIA.^1,2^

## Pathogenesis

The *PANK2* gene is responsible for production of the pantothenate kinase 2 enzyme, which regulates the formation of coenzyme A (CoA). The deficiency of CoA is supposed to increase synthesis of oxygen free radicals, eventually leading to destruction of the phospholipid membrane, especially in the basal ganglia and retina, followed by iron accumulation.^1^

Iron accumulation leads to the characteristic rust-brown discolouration and this is why the disease is sometimes called pigmentary degeneration of the GP. Generalized atrophy may be noticed. Microscopic features include loss of neurons with gliosis, vacuoles and presence of spheroids, which are non-nucleated structures that represent swollen axons.^1^

## Clinical presentation

The two main forms of clinical presentations are a classical form, which involves a rapid progression of symptoms that develop in the first decade of life, and an atypical form, which, by comparison to the classical form, is slowly progressive and commences after the age of 10 years. Symptoms include dystonia, dysarthria, rigidity, choreoathetosis and cognitive decline. Visual impairment may occur owing to pigmentary retinopathy.^1,3^

## Imaging findings

The use of MRI for diagnosis was first reported by Tanfani et al.^4^ Owing to accumulation of iron, *T*_2_ weighted images demonstrate hypointense signal in GP and substantia nigra (SN). Owing to tissue gliosis and vacuolization in the medial aspect of the GP, a central hyperintensity appears medially within the hypointensity. This appearance was described by Sethi as the “eye-of-the-tiger” sign.^2^ The decreased signal owing to iron accumulation is more obvious in GRE and susceptibility weighted imaging (SWI) sequences. No diffusion restriction or enhancement occurs. Diffusion tensor imaging shows increased fractional anisotropy in GP and SN and MR spectroscopy shows decreased *N*-acetylaspartate (NAA) and reduced NAA:creatinine ratio owing to neuroaxonal loss.^5^

## Differential diagnosis

Physiological iron accumulation in the basal ganglia shows decreased signal in SWI and GRE sequences, but it never occurs before the age of 10 and usually after the age of 25 years.^1^ Other types of NBIA should be considered but differentiation, especially early in the disease process, is difficult using MRI only. Specific radiological features in other types of NBIA include cerebellar atrophy in PLA2G6-associated neurodegeneration; cortical atrophy in mitochondrial-membrane protein-associated neurodegeneration; *T*_1_ hyperintense signal within the hypointensity in SN in beta-propeller protein-associated neurodegeneration; pontocerebellar atrophy in fatty acid hydroxylase-associated neurodegeneration and *T*_2_ hyperintense signal in putamen and thalami with GP calcification in COASY protein-associated neurodegeneration.^3^

Other metabolic disorders, such as Wilson’s disease, Leigh disease and infantile bilateral striatal necrosis, show hyperintensity, predominantly in the caudate and putamen and not the GP.^5^ In a study of 123 patients with PKAN, every patient with mutation in *PANK2* gene was found to have the eye-of-the-tiger sign on MRI, and therefore the eye-of-the-tiger sign is reported to have one-to-one correlation with *PANK2* mutation.^2^ However, the sign may be found in other diseases, such as cortical basal ganglionic degeneration, early-onset levodopa responsive parkinsonism and progressive supranuclear palsy.^6^ The sign has also been reported in a healthy individual.^7^

## Diagnosis

The presence of severe *T*_2_ shortening and blooming on *T*_2_* sequences in a child should raise the suspicion of NBIA. Not every case will show the eye-of-the-tiger sign.^5^ In the early course of the disease, isolated hyperintensity without the full picture involving the presence of the eye-of-the-tiger may be observed. In these cases, with the presence of suggestive clinical features, genetic testing is necessary. The complete MRI features may even precede the clinical symptoms.^8^

## Treatment

As yet, there is no definitive treatment. The management has been principally symptomatic. Treatments under investigation include iron-chelating agents that cross the blood–brain barrier and deep brain stimulation of the GP.^3^

In 1992, Okano^9^ described a case of Down syndrome in which progressive neurological deficit occurred with the morphological picture of Hallervorden–Spatz syndrome on MRI. To our knowledge, our case is the first one describing genetically proven PKAN in a patient with mosaic trisomy 21.

The imaging features in our patient are characteristic of PKAN. Although basal ganglion changes are reported in patients with Down syndrome, in our case, there is no evidence of contribution of the trisomy 21 mosaicism in the imaging features. The simultaneous presence of these two different genetic conditions in a single patient gives this case its remarkable medical value.

## Learning point

Although the eye-of-the-tiger sign, in the appropriate clinical settings, is indicative of PKAN, it may develop at a variable point in the disease process. The presence of uncertain bilateral changes in the basal ganglia in an infant with symptoms suggestive of the disease should be further investigated.
